# VISTA regulates microglia homeostasis and myelin phagocytosis, and is associated with MS lesion pathology

**DOI:** 10.1186/s40478-021-01186-7

**Published:** 2021-05-18

**Authors:** Malte Borggrewe, Susanne M. Kooistra, Evelyn M. Wesseling, Fenja L. Gierschek, Maaike L. Brummer, Elizabeth C. Nowak, Tiago Medeiros-Furquim, Tegan A. Otto, Sam W. Lee, Randolph J. Noelle, Bart J. L. Eggen, Jon D. Laman

**Affiliations:** 1 Department of Biomedical Sciences of Cells and Systems, Section Molecular Neurobiology, University Medical Center Groningen, University of Groningen, and MS Centrum Noord Nederland (MSCNN), Groningen, The Netherlands; 2 Department of Microbiology and Immunology, Geisel School of Medicine at Dartmouth, Norris Cotton Cancer Center, Lebanon, NH USA; 3 Yale University School of Medicine, New Haven, CT USA

**Keywords:** Immunotherapy, Neurodegeneration, Glial cells, Demyelination, PD-1H, Checkpoint inhibition

## Abstract

V-type immunoglobulin domain-containing suppressor of T-cell activation (VISTA) is a negative checkpoint regulator (NCR) that is involved in T-cell quiescence, inhibition of T-cell activation, and in myeloid cells regulates cytokine production, chemotaxis, phagocytosis, and tolerance induction. In the central nervous system (CNS), VISTA is expressed by microglia, the resident macrophage of the parenchyma, and expression is decreased during neuroinflammation; however, the function of VISTA in microglia is unknown. Here, we extensively analyzed VISTA expression in different MS lesion stages and characterized the function of VISTA in the CNS by deleting *VISTA* in microglia. VISTA is differentially expressed in distinct MS lesion stages. In mice, *VISTA* deletion in Cx3Cr1-expressing cells induced a more amoeboid microglia morphology, indicating an immune-activated phenotype. Expression of genes associated with cell cycle and immune-activation was increased in VISTA KO microglia. In response to LPS and during experimental autoimmune encephalomyelitis (EAE), VISTA KO and WT microglia shared similar transcriptional profiles and *VISTA* deletion did not affect EAE disease progression or microglia responses. VISTA KO in microglia in vitro decreased the uptake of myelin. This study demonstrates that VISTA is involved in microglia function, which likely affects healthy CNS homeostasis and neuroinflammation.

**Supplementary Information:**

The online version contains supplementary material available at 10.1186/s40478-021-01186-7.

## Introduction

Immune checkpoints are a group of receptors and ligands expressed by antigen-presenting cells (APC) and T cells that regulate T-cell activation by providing costimulatory and coinhibitory signals. These signals are essential for balancing immunity to allow response to pathogens while limiting the risk of developing autoimmunity. Negative checkpoint regulators (NCR) are involved in inhibition of T-cell activation and blocking their activity using immune checkpoint inhibitors is an effective treatment against cancer [10, 15, 26].

Cancer treatment with immune checkpoint inhibitors (immunotherapy) can have adverse effects on the central nervous system (CNS) since patients are susceptible to developing paraneoplastic syndrome, demyelination, encephalitis, multiple sclerosis (MS), and hypophysitis [14, 50]. Furthermore, blocking the NCR PD1 in mouse models of Alzheimer’s disease (AD) may alleviate the disease [3], although this concept is disputed [34]. Thus, determining NCR function in the CNS will provide valuable insight into how adverse effects of immunotherapy could be limited, and whether NCR modulation can be used as a therapeutic strategy against CNS diseases such as AD and MS.

V-type immunoglobulin domain-containing suppressor of T-cell activation (VISTA) is an NCR predominantly expressed on myeloid- and T cells which is involved in T-cell quiescence and inhibition of T-cell activation [19, 20, 47]. In addition to its role as an NCR, VISTA has multiple functions in monocytes/macrophages including the phagocytosis of apoptotic cells (efferocytosis) [13, 49], cytokine production [4, 5], tolerance induction [18], and chemotaxis [9, 41].

Recently, we have demonstrated that in the CNS VISTA is predominantly expressed by microglia and to lesser extent by endothelial cells [6]. Microglia are the main resident myeloid cells of the CNS and continuously scan their environment for perturbations and intruders. They are involved in maintaining homeostasis, for example, by clearing cell debris, and are functionally implicated in virtually all CNS diseases [39]. Microglial VISTA expression decreases after toll-like receptor (TLR) ligation (TLR 1–4, 6) in vitro, lipopolysaccharide (LPS) exposure in mice, and during murine experimental autoimmune encephalomyelitis (EAE), a model for MS [6]. In post-mortem tissue of an MS patient, VISTA expression was decreased in chronic active lesions compared to normal-appearing white matter (NAWM) [6]. Furthermore, VISTA is differentially regulated in numerous other CNS inflammatory diseases, reviewed in [7].

The function of VISTA in microglia and the CNS during health and disease remains to be elucidated [7]. To that end, we deleted *VISTA* from Cx3cr1-expressing cells to assess the function of VISTA in microglia during homeostasis and neuroinflammation, after an LPS challenge and in EAE. These mouse experiments were complemented by the detailed characterization of VISTA expression in distinct MS lesion stages in human brain tissue.

## Materials and methods

### Mice

Animal experiments were approved by the Netherlands Central Committee for Animal Experiments and the University of Groningen. Mice were housed specific-pathogen free in macrolon cages, a 12 h light–dark cycle, and with ad libitum access to food and water unless specified otherwise. Conditional VISTA KO mice (Cx3cr1^creERT2/WT^ VISTA^loxP/loxP^) were generated by crossbreeding VISTA^loxP/loxP^ [49] and B6.129P2(Cg)-Cx3cr1^tm2.1(cre/ERT2)Litt^/WganJ mice (Jax, 021,160). Genotype was confirmed (see below) before depletion of VISTA in conditional VISTA KO mice by administration of 20 mg tamoxifen (Sigma-Aldrich, T5648) via oral gavage and subsequent 5-week rest to allow turnover of peripheral CX3CR1^pos^ cells (= VISTA KO). Littermate controls (Cx3cr1^creERT2/WT^ VISTA^loxP/loxP^) treated with corn oil served as control (= VISTA WT). For LPS experiments, 10-week-old conditional VISTA KO mice were injected with 1 mg/kg LPS (*E. coli*, O111:B4, L4391) intraperitoneally and terminated 3 h later. PBS-injected mice served as control. For EAE experiments, 11-week-old female conditional VISTA KO mice were immunized with MOG_35-55_ in complete Freund’s adjuvant in the neck and lower back according to manufacturer’s instructions (Hooke, EK-2110). At the time of immunization and after 24 h, mice were injected intraperitoneal (i.p.) with 150 ng pertussis toxin intraperitoneally. Animals were scored daily for disease symptoms and terminated at score 1 (limp tail; early disease), score 4 (hind leg paralysis; peak disease), and chronic (3 weeks after onset of symptoms). Unimmunized mice served as control.

### Genotyping

Genotype for experimental animals (Cx3cr1^creERT2/WT^ VISTA^loxP/loxP^) was confirmed by genomic PCR for *Cx3cr1* WT and creERT2 alleles, and for *VISTA*. Genomic DNA was extracted from earcuts of mice using MyTaq Extract-PCR kit (Bioline, BIO-21127) according to manufacturer’s instructions. Genes and alleles were amplified using MyTaq HS Red Mix (Bioline, BIO-25047) and 10 μM primer and analyzed on 2% agarose gels. For *Cx3cr1* WT and creERT2 alleles, two primer-pairs were used for WT allele (fwd: 5′-CTCAC GTGGA CCTGC TTACTG; rev: 5′-GTACC GGTCG ATGCT GATGA) and creERT2 allele (fwd: 5′-AAGAC TCACG TGGAC CTGCT; rev: 5′-CGGTT ATTCA ACTTG CACCA) with the following PCR program: 1) 95 °C 3 min, 2) 95 °C 15 s, 3) 58 °C 15 s, 4) 72 °C 20 s, repeated 30 times. *Cx3cr1* WT alleles present with a band at 482 bp, whereas *Cx3cr1* creERT2 alleles present with a band at 260 bp. For *VISTA*, one primer pair was used (fwd: 5′-CTAAT GGCAC AGCAG GGACT; rev: 5′-CAACA AATCA CGGTG GAGTG) with the following PCR program: 1) 95 °C 3 min, 2) 95 °C 15 s, 3) 51.7 °C 30 s, 4) 72 °C 30 s, repeated 35 times. *VISTA* WT alleles present with a band at 468 bp, whereas *VISTA* loxP alleles present with a band at 651 bp.

### Microglia isolation

Microglia were isolated from whole brain (LPS) or spinal cord (EAE) as previously described in detail [21]. The whole isolation procedure was performed on ice. Mice were perfused with PBS (Lonza, BE17-512F) and CNS tissue was mechanically dissociated in HBSS (Gibco, 14,170–088) containing 0.6% glucose (Sigma-Aldrich, G8769) and 15 mM HEPES (Lonza, BE17-737E). Myelin was removed by 24.4% percoll (GE Healthcare, 17-0891-01) density gradient centrifugation at 950 g for 20 min at 4 °C. Fc receptors were blocked with anti-CD16/32 (5 μg/ml, clone 93, eBioscience, 14-0161-85), and cells were stained with anti-CD11b-APC-Cy7 (1 μg/ml, clone M1/70, eBioscience, A15390), anti-CD45-PE-Cy7 (1 μg/ml, clone 30-F11, eBioscience, 25-0451-82), anti-Ly6C-APC (1.5 μg/ml, clone HK1.4, Biolegend, 128,016), and anti-VISTA-PE (20 μg/ml, clone MIH63, Biolegend, 150,204) antibodies 30 min at 4 °C in HBSS without phenol red (Gibco, 14,175-053) containing 0.6% glucose, 15 mM HEPES, and 1 mM EDTA (Invitrogen, 15,575-020). Microglia were sorted on a MoFlo Astrios (Beckman Coulter) in siliconized tubes containing RNAlater (Qiagen, 76,104), centrifuged at 5000 g, and lysed in RLT + lysis buffer (Qiagen, 74,034).

### RNA extraction and sequencing

RNA was extracted using AllPrep DNA/RNA Micro Kit (Qiagen, 80,284) according to manufacturer’s instructions. Total RNA concentration was measured on TapeStation 4200 and samples with a RIN value below 7 were excluded. Sequencing libraries were generated using a NEBNext single cell/low input RNA library prep kit for Illumina (NEBNext, E6429), which was modified to include unique molecular identifiers for removal of PCR duplicates. Libraries were sequenced at 20 million read depth on an Illumina NovaSeq6000.

### Bioinformatic analysis

#### Alignment

After removal of low-quality reads and adapter sequences, reads were mapped to the mouse GRCm38.p6 genome using Tophat (v2.0.14) [30] with default settings. Feature counting to quantify gene expression was done using HTSeq (v0.11.0) [1].

#### Differential gene expression analysis

Low expressed genes with no expression in at least two samples were excluded before DESeq2 R-package (v1.30.0) [37] was used for transformation, normalization, and differential gene expression analysis. Two samples were excluded based on low library size and complexity. Variance stabilizing transformation of counts was done for principal component analysis (PCA). Genes were regarded differentially expressed with a log2 Fold change > 1 and a Benjamini–Hochberg adjusted *p*-value of < 0.05. Additional file 1: Tables S4 and S5 provide normalized expression of genes for each sample and results of differential gene expression analysis for LPS and EAE experiments.

#### Weighted gene co-expression network analysis (WGCNA)

After filtering of low expressed genes as above, variance stabilizing transformed counts were used as input for WGCNA (v1.69) [33]. Genes with zero variance and missing values were excluded before constructing a signed network using dissimilarities of topological overlap matrix. Modules with a minimum size of 30 genes were constructed and merged with a threshold of 0.25. Module trait correlation was regarded significant with a *p*-value < 0.05.

#### Enrichment for biological processes and transcription factors

To determine potential biological processes and transcription factors associated with genes in WGCNA modules and differentially expressed genes, enrichr (v3.0) [12] was used to perform an enrichment analysis for gene ontology biological processes, molecular signatures database hallmarks, and ENCODE/ChEA transcription factor targets.

### Primary neonatal mouse microglia culture

Primary neonatal mouse microglia cultures were generated from P0-3 pups as previously described [6, 40]. After removal of meninges and cerebellum, the cerebrum was minced and incubated in HBSS containing 0.6% glucose, 1 × DNase (Sigma-Aldrich, DN25), 0.25% trypsin (Lonza, BE02007E), 1% penicillin–streptomycin (Gibco, 15,140,122), and 15 mM HEPES (Lonza, BE17-737E) for 20 min. The solution was mechanically dissociated further and centrifuged at 230 g for 10 min. Cells were plated in flasks in DMEM (Gibco, 1,500,416) containing 1 mM sodium pyruvate (Lonza, BE13-115E), 1 × GlutaMAX (Gibco, 35,050,038), 1% penicillin–streptomycin, and 10% FCS (Gibco, 10,500,064) (microglia medium). Medium was replaced after 1, 4, and 6 days. After 9 days, medium was replaced with fresh medium containing 33% L929 cell-conditioned medium that contains M-CSF to stimulate microglia proliferation. Microglia were harvested after 2 days by mitotic shake-off and seeded at 30,000 cells/cm^2^ in experimental plates. For deletion of VISTA, primary microglia cultures were prepared from pups of Cx3cr1^creERT2/WT^ VISTA^loxP/loxP^ mice. After mitotic shake-off, microglia were treated with 0.1 μM (Z)-4-hydroxytamoxifen (Sigma-Aldrich, H7904) or 0.01% ethanol for 24 h and left to rest for 48 h in microglia medium. Subsequently, microglia were used for phagocytosis assay (see below) or stimulated for 5 h with 100 ng/ml Pam3CSK4 (Invivogen, #tlrl‐pms), 100 ng/ml LPS (*E. coli* 0111:B4, Sigma‐Aldrich, L4391), 50 μg/ml polyI:C (Invivogen, #tlrl‐pic), 10 μg/ml beta-glucan (Sigma-Aldrich, G5011), 10 ng/ml IL1b (Peprotech, 400-18), 10 ng/ml TNFalpha (Peprotech, 400-14), 10 ng/ml IL10 (Peprotech, 400-19), 20 mg/ml IL4 (Peprotech, 400-04), 10 ng/ml TGFbeta (Peprotech, 100-16), or mouse myelin (see below).

### Induction of early apoptotic Jurkat cells

Jurkat human T cell line was kindly provided by Prof. J. Smit (UMCG) and cultured in RPMI (Gibco, 21,875,034) supplemented with 10% FBS, 1 × penicillin–streptomycin, and 1 mM sodium pyruvate. Apoptosis induction for phagocytosis assay (described below) was done on the day of performing the assay as previously described [2]. Jurkat cells were collected at 1*10^6^ cells/mL in medium and treated with 1 μM staurosporine (Sigma-Aldrich, S5921) for 4 h. To verify induction of apoptosis, Jurkat cells were labelled with Annexin V/propidium iodide (Biolegend, 640,914) according to manufacturer’s instructions and analyzed on a MacsQuant (Miltenyi Biotec). After 4 h of staurosporine exposure, > 90% of cells were early apoptotic (Additional file 7: Fig. S6D).

### Myelin isolation

Myelin from 10 week old C57BL/6 mouse whole brains was isolated as described previously [38] with minor adjustments. Whole brains were mechanically dissociated in HBSS containing 0.6% glucose and 15 mM HEPES and centrifuged in 24.4% percoll at 950 g for 20 min. The upper layer containing myelin was collected, diluted 1:3 in HBSS, and centrifuged at 950 g for 15 min. The pellet was resuspended in 0.32 M sucrose and a layer of 0.85 M sucrose was carefully added on top. The solution was centrifuged at 75,000 g for 30 min at 4 °C and the cloudy interphase between the two sucrose layers was collected and resuspended in Milli-Q water. After centrifugation at 75,000 g for 30 min at 4 °C, the pellet was washed twice by resuspending in Milli-Q water and centrifuging at 13,500 g for 15 min at 4 °C. As before, the pellet was resuspended in 0.32 M sucrose and 0.85 M sucrose was added on top before centrifuging at 75,000 g for 30 min at 4 °C and collecting the cloudy interphase. The interphase was diluted in Milli-Q and centrifuged at 75,000 g for 15 min at 4 °C and the remaining pellet containing pure myelin was resuspended in 1 mL sterile PBS. The Pierce BCA protein assay kit (Thermo Scientific, 23,225) was used to determine the myelin concentration.

### Phagocytosis assay

Fresh early apoptotic Jurkat cells (EAJ) and mouse myelin were labelled with pHrodo Red succinimidyl ester (Invitrogen, P36600) according to manufacturer’s instructions. Briefly, 10*10^6^ EAJ cells/mL or 1 mg/mL mouse myelin was mixed with 0.1 mg/mL pHrodo Red succinimidyl ester and incubated for 1 h at RT with intermittent mixing. Labelled compounds were diluted in cold PBS and centrifuged before resuspending the pellet in DMEM containing 1 mM sodium pyruvate, 1 × GlutaMAX and 1% penicillin–streptomycin (quiescence medium).

Primary neonatal mouse microglia were stimulated with LPS or PBS 5 h prior to the addition of phagocytosis compounds. Microglia were pretreated with 10 μM cytochalasin D (Sigma-Aldrich, C8273) to inhibit phagocytosis or with 0.1% DMSO before adding 20 μg/mL pHrodo-labelled myelin, 20 μg/mL pHrodo red *E. coli* BioParticles (Invitrogen, P35361), and pHrodo-labelled EAJ at equal numbers as seeded microglia. PHrodo signal was detected every 15 min over 12 h using an IncuCyte Live Cell Analysis System (Sartorius).

### RT-qPCR

Total RNA from primary neonatal mouse microglia was isolated using TRIzol (Invitrogen, 15,596,018) and cDNA was generated using RevertAid First Strand cDNA Synthesis Kit (ThermoFisher, K1622) according to manufacturer’s instructions. Quantitative PCR was done using iTag Universal SYBR Green Super-Mix (Bio-Rad, 1,725,125) and exon-exon spanning primers on a QuantStudio 7 Flex (ThermoFisher). Primer pairs used were for *VISTA* (fwd: 5′- AACAA CGGTT CTACG GGTCC; rev: 5′-CGTGA TGCTG TCACT GTCCT), *Tnf* (fwd: 5′- TCTTC TGTCT ACTGAA CTTCGG; rev: 5′- AAGAT GATCT GAGTGT GAGGG), *Ccl2* (fwd: 5′-TCAGC CAGAT GCAGT TAACG; rev: 5′-CTGGT GATCC TCTTG TAGCTC), and *Hprt1* (fwd: 5′-ATACA GGCCA GACTTT GTTGGA; rev: 5′-TGCGC TCATCT TAGGC TTTGTA).

### Flow cytometry

Primary neonatal mouse microglia were detached using accutase (Sigma-Aldrich, A6964) for 10 min at 37 °C and resuspended in HBSS without phenol red containing 15 mM HEPES, 0.6% glucose, and 1 mM EDTA. Microglia were blocked using anti-CD16/32 (5 μg/ml) for 15 min at 4 °C and stained with anti-VISTA-PE (20 μg/ml) for 30 min at room temperature (RT). Microglia were analyzed on a MacsQuant (Miltenyi Biotec).

### Immunohistochemistry

Immunohistochemical staining was performed on formalin-fixed paraffin-embedded (FFPE) (human) or paraformaldehyde-fixed frozen (FF) (mouse) tissue. FFPE tissue was deparaffinized in xylene (J.T. Baker, 9490) and rehydrated. FF tissue was dried in an exsiccator. Heat-induced epitope retrieval was performed in a microwave in sodium citrate (pH = 6.0) using a pressure cooker. Endogenous peroxidase activity was blocked for enzymatic immunohistochemistry using 0.3% hydrogen peroxide for 30 min. Mouse tissue was additionally blocked 1 h in 5% normal serum. Antibodies were diluted in PBS containing 0.1% Triton-X (mouse) and 1% normal serum, or in Normal Antibody Green Bright Diluent (human) (ImmunoLogic, BD09-500). Antibodies used were anti-VISTA (1:200, clone D1L2G, Cell Signaling, #64,953), anti-IBA1 (1:1000, polyclonal, Wako, 019-19,741), anti-HLA-DR (1:750, clone LN3, eBioscience, 14-9956-82), anti-PLP (1:500, clone plpc1, Bio-Rad, MCA839G), anti-CD68 (1:500, clone PG-M1, Dako, M0876), and anti-TMEM119 (1:500, polyclonal, Atlas Antibodies, HPA051870). Biotinylated secondary antibodies (1:400, Vector, BA-1000 and BA-2001) were applied for 1 h at RT. Vectastain Elite ABC-HRP (Vector, PK-6100) was applied and immunoreactivity was revealed using 3,3′-diaminobenzidine (DAB).

### Microglia morphological analysis

Mouse tissue was stained with anti-IBA1 and slides were scanned on a NanoZoomer Digital Pathology System (Hamamatsu Photonics) with a 40 × objective. An analysis tool was developed [27, 51] to evaluate 27 microglia morphological features. Briefly, images of at least 20 randomly selected individual microglia in cortex per mouse were processed to obtain cell silhouette images using semi-automated thresholding. Following thinning and pruning of branch areas to obtain cell skeletons, branch endings (end nodes), branch crossings (junctions) and all branchpoints going out from the cell soma (start nodes) were marked. Cell silhouettes and skeletons were used for fully automated morphological analysis including Sholl analysis and 23 other morphometric features. A non-supervised clustering algorithm was used to identify microglia subpopulations based on morphology. To this end, morphometric features were normalized and scaled before reducing the dimensionality using principal component analysis (PCA). Hierarchical clustering using Ward’s method was used to determine the top-contributing PC with an eigenvalue > 1. Morphometric features of individual cells are provided in Additional file 1: Table S2.

### Statistical analysis

Statistical analyses were performed using GraphPad Prism (v8.4.0). The type of statistical test used is indicated in the figure legends. Values of *p* < 0.05 were considered significant: *p < 0.05, **p < 0.01, ***p < 0.001, ****p < 0.0001.

## Results

### VISTA is differentially expressed in distinct MS lesion stages and decreased during inflammation

We previously demonstrated that VISTA expression is decreased in a chronic active lesion of an individual MS patient sample, and in microglia during inflammation in vivo and in vitro [6]. To expand on these observations, we analyzed VISTA expression in a comprehensive set of 14 post-mortem tissues from MS patients supplied by the Netherlands Brain Bank (Additional file 1: Table S1), covering all lesion stages, including white and gray matter lesions.

MS post-mortem tissues were staged based on the degree of demyelination using proteolipid protein (PLP) and inflammation using human leukocyte antigen DR isotype (HLA-DR, MHC-II) [32, 43, 44] and contained 6 different MS lesion stages (Additional file 2: Fig. S1). Normal-appearing white matter (NAWM) are areas in post-mortem tissue without overt evidence of inflammation and demyelination (Additional file 2: Fig. S1). Preactive lesions show microglia activation (based on HLA-DR expression), but no demyelination, and active lesions are demyelinating and contain many HLA-DR positive cells (Additional file 2: Fig. S1). Chronic active lesions are divided into a center area, which is demyelinated without aberrant inflammation, and a rim, which is not demyelinated but shows inflammation (Additional file 2: Fig. S1). Inactive lesions present with demyelination but no inflammation, whereas remyelinated lesions, or shadow plaques, are partially remyelinated (Additional file 2: Fig. S1). Cortical lesions are similar to active lesions, but are located in gray matter (GM) (Additional file 2: Fig. S1).

**Fig. 1:**
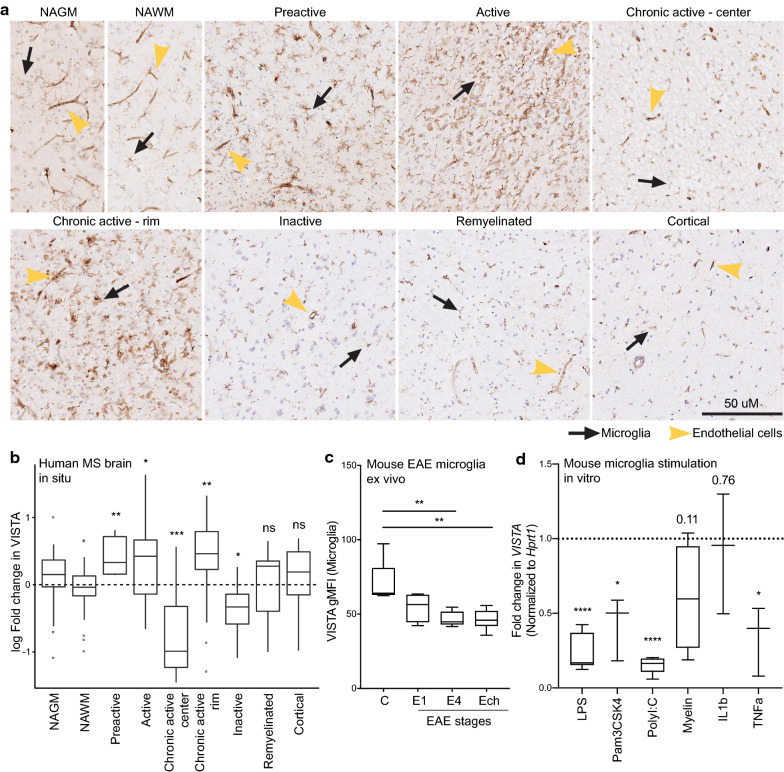
VISTA is differentially expressed in distinct MS lesion stages and decreases in EAE. **a** Representative images of in situ expression of VISTA in distinct MS lesion stages. **b** Quantification of the area positive for VISTA in MS lesion stages compared to normal-appearing regions shown as log fold change. At least 3 images were taken from each lesion and the area positive for VISTA was measured. Statistical analysis performed was a Wilcoxon signed-rank test comparing lesions to normal-appearing areas with Benjamini–Hochberg correction for multiple comparisons (n = 5 for each lesion). (c) VISTA geometric mean fluorescence intensity (gMFI) on viable (DAPIneg) microglia (CD45intCD11BposLY6Cneg) during different stages of EAE including score 1(E1: early disease) score 4 (E4: peak disease), and chronic EAE (Ech), and unimmunized control (c) mice. Error bars indicate mean ± s.d. (n = 5–6). Statistical analysis performed was a one-way ANOVA with Dunnett’s correction for multiple comparisons. d Fold change in VISTA expression in primary neonatal mouse microglia 5 h after stimulation with LPS, Pam3CSK4, PolyI:C, myelin, IL1b, or TNFa, measured by RT-qPCR and normalized to Hprt1. Error bars indicate mean ± s.d. (n = 3–5). Statistical analysis performed was a one-way ANOVA with Dunnett’s correction for multiple comparisons. Boxes indicate median and lower and upper quartiles, whiskers show min/max, and outliers are indicated by points. ns = not significant, *p < 0.05, **p < 0.01, ***p < 0.001, ****p < 0.0001

To assess VISTA expression in MS lesions, immunohistochemical stainings were quantified using the area positive for VISTA (n = 5 patients for each lesion). VISTA was expressed in all different MS lesion stages by microglia and endothelial cells to varying degrees (Fig. 1a, b). In preactive, active, and the rim of chronic active lesions, VISTA expression was increased, whereas VISTA expression was decreased in inactive and the center of chronic active lesions compared to NAWM (Fig. 1a-b). No change was detected in remyelinated or cortical lesions compared to NAWM and NAGM, respectively (Fig. 1a–b). In addition, expression of microglia markers IBA1, TMEM119, and the immune-activation/phagocytosis marker CD68 were analyzed in MS lesions (Additional file 3: Fig. S2A-F). IBA1 was increased in preactive and in the rim of chronic active lesions, whereas it was decreased in inactive and the center of chronic active lesions (Additional file 3: Fig. S2A + D). TMEM119 expression was increased in the rim of chronic active lesions, but decreased in active, inactive, and the center of chronic active lesions, consistent with previous studies [45, 52] (Additional file 3: Fig. S2B + E). CD68 expression was increased in preactive, active, and the rim of chronic active lesions, whereas it was decreased in inactive lesions. Linear regression analysis revealed that VISTA expression correlated mostly with expression of microglia markers IBA1 and TMEM119, and it did not strongly correlate with inflammatory markers HLA-DR and CD68 (Additional file 3: Fig. S2C + F). These findings may indicate that VISTA in MS lesions is expressed by microglia rather than pro-inflammatory immune cells.

Next, we analyzed VISTA expression by microglia at different stages of EAE including score 1 (E1; early disease), score 4 (E4; peak disease), and chronic EAE (Ech), and unimmunized mice served as a control (C) (Fig. 1c). At all three EAE stages, surface VISTA expression was decreased (Fig. 1c), corroborating our previous findings of reduced *VISTA* mRNA expression during EAE [6]. To address which inflammatory mediators may be responsible for a decreased VISTA expression in EAE and differential VISTA expression in different MS lesion stages, primary neonatal mouse microglia were stimulated with several pro-inflammatory compounds (Fig. 1d). In response to TLR ligands LPS (TLR4), Pam3CSK4 (TLR1/2), and polyI:C (TLR3), *VISTA* expression was decreased in microglia in vitro, which verifies our previous observations [6]. *VISTA* mRNA expression was also decreased after stimulation with tumor necrosis factor alpha (TNFa), but not interleukin (IL) 1 beta, and a near-significant reduction in VISTA levels was observed after stimulation with mouse myelin (Fig. 1d).

These findings demonstrate that VISTA is differentially expressed in distinct MS lesion stages. Furthermore, VISTA is decreased in microglia at all stages of EAE, as well as after stimulation with various pro-inflammatory compounds in microglia in vitro indicating that VISTA expression is dynamically regulated under inflammatory conditions.

### VISTA KO reduces microglia ramification in PBS but not LPS-treated mice

**Fig. 2:**
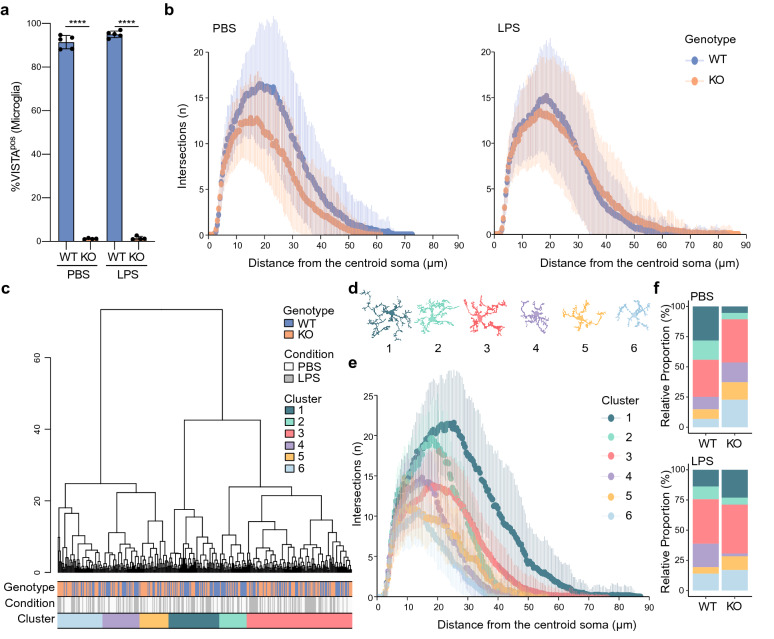
VISTA KO reduces microglia ramification in PBS but not LPS-treated mice. (A) Percentage VISTA^pos^ microglia (DAPI^neg^ CD11b^pos^ CD45^int^ Ly6C^neg^) in WT and VISTA KO whole brain microglia after intraperitoneal PBS or LPS injection. Error bars indicate mean ± s.d. (n = 5). Statistical analysis performed was a one-way ANOVA with Bonferroni correction for multiple comparisons. ****p < 0.0001 **b** Sholl analysis of WT and VISTA KO microglia after PBS (left) and LPS injection (right) (n = 25 microglia per mouse, n = 3 mice per condition). **c** Hierarchical clustering on principal components of 27 morphological features and corresponding genotype, condition, and cluster per cell. **d** Representative cell per cluster visualized as cell silhouettes. **e** Sholl analysis of microglia from each cluster. **f** Frequency of cells per cluster in WT and VISTA KO mice after PBS and LPS injection

VISTA is decreased in microglia after LPS injection, during EAE, and in neurodegenerative disease models in mice [6, 7]. To investigate the role of VISTA in microglia, *VISTA* was deleted from Cx3cr1-expressing cells using Cx3cr1^creERT2/WT^ VISTA^loxP/loxP^ mice. Tamoxifen induced stable and efficient deletion of *VISTA* and reduced mRNA and surface VISTA expression in microglia after 1.5, 3, and 6 months, and in PBS and LPS-treated mice (Additional file 4: Fig. S3A-C). The percentage of VISTA-expressing microglia (DAPI^neg^ CD11b^pos^ CD45^int^ Ly6C^neg^) was reduced from > 90% in oil-treated (= VISTA WT) to ~ 1% in tamoxifen-treated mice (= VISTA KO) (Fig. 2a).

Next, we analyzed the effect of VISTA KO on microglia morphology in cortex of PBS-injected mice and after intraperitoneal LPS injection using Sholl analysis and assessment of 23 other morphological parameters [27, 51] (Additional file 1: Table S2). Alterations in microglia morphologies are associated with CNS disease and underlying changes in microglia phenotypes [16]. Sholl analysis revealed that VISTA KO microglia exhibited a less ramified morphology compared to VISTA WT microglia in PBS mice (Fig. 2b). LPS treatment reduced the ramification in WT microglia, but not in VISTA KO microglia (Fig. 2b). To further characterize microglia morphologies, individual cells were clustered based on principal component analysis of all morphological parameters, resulting in 6 distinct clusters (Fig. 2c). Microglia in these clusters showed distinct morphologies reflected by deviating morphological features such as ramification (Fig. 2d–e). In PBS-treated VISTA KO mice, cluster 1–2 microglia were underrepresented compared to WT, whereas cluster 3–6 microglia were enriched (Fig. 2f). Cluster 1 and 2 microglia were highly ramified, whereas cluster 3–6 microglia exhibited a more amoeboid morphology (Fig. 2d–e). After LPS injection, differences in cluster distribution were less apparent in VISTA KO compared to WT microglia, and cluster 4 microglia, which showed intermediate ramification (Fig. 2d), were almost exclusively detected in WT (Fig. 2f).

This analysis revealed that in PBS-treated mice VISTA KO microglia exhibit reduced ramification compared to WT, but that ramification is not further reduced by LPS, indicating that VISTA regulates microglia homeostasis, but not the LPS response.

### VISTA KO induces an immune-activated and proliferative microglia transcriptional profile in naive mice, but does not affect the LPS response

Since VISTA regulates microglia morphology in PBS-treated mice but does not seem to affect the microglia LPS response based on morphology, we next investigated genome-wide transcriptional changes in VISTA KO and WT brain microglia in PBS-treated mice and 3 h after an i.p. LPS injection.

**Fig. 3:**
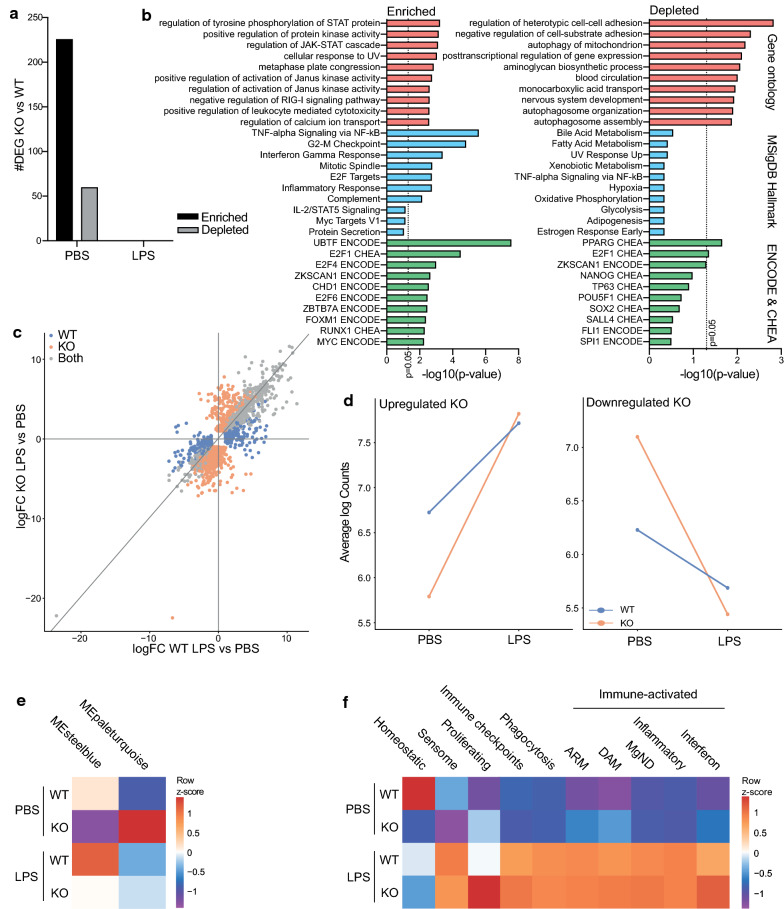
VISTA KO microglia in PBS-treated mice are transcriptionally distinct but not after LPS. **a** Number of differentially expressed genes (DEGs; logFC > 1; adjusted *p*-value < 0.05) comparing VISTA KO vs WT microglia from whole brain after PBS and LPS injection. **b** Gene ontology biological processes (red bars), molecular signatures database hallmark (blue bars), and ENCODE/CHEA transcription factors (green bars) associated with genes enriched (left) and depleted (right) in VISTA KO microglia compared to WT in PBS-treated mice. **c** Four-way plot illustrating logFC of DEGs after LPS compared to PBS in VISTA WT and KO mice. Color of dots indicates whether genes are differentially expressed (adjusted p-value < 0.05) in WT (blue), KO (orange), or both (grey). **d** Average expression of genes uniquely up- (left) or downregulated (right) in VISTA KO microglia after LPS across all samples. **e** Expression of Eigengenes of gene modules significantly correlating with genotype identified by WGCNA illustrated as row z-score. **f** Average expression of published gene sets associated with distinct microglia functions across all samples [24, 28, 29, 31, 42]. Additional information on gene sets can be found in Additional file 1: Table S3. logFC = log2 fold change, ARM = activated response microglia, DAM = disease-associated microglia, MgND = microglia neurodegenerative phenotype

In PBS-treated mice, 286 (226 enriched; 60 depleted) genes were differentially expressed (log fold change > 1, *p*-adjusted < 0.05) between VISTA KO and WT microglia (Fig. 3a). Genes enriched in VISTA KO microglia were associated with immune signaling such as JAK-STAT, TNFa, and interferon gamma (IFNg), and with cell cycle processes (E2F targets, G2-M checkpoint) (Fig. 3b). Genes depleted in VISTA KO microglia compared to WT were annotated with processes of cell–cell and cell-substrate adhesion and autophagosomes (Fig. 3b).

LPS injection led to a typical microglia LPS-response in both WT and VISTA KO microglia, and no difference between KO and WT was observed in the level of induction of our previously published microglia LPS response gene set [22] (Additional file 5: Fig. S4A-B). Concordantly, there were no differentially expressed genes (DEGs; log2 fold change, logFC > 1, p-adjusted < 0.05) after LPS treatment between WT and VISTA KO microglia (Fig. 3a). Similar observations were made in vitro, since stimulation of VISTA KO primary neonatal mouse microglia with various inflammatory stimulants did not alter the induction of *Tnf* or *Ccl2* compared to WT microglia (Additional file 5: Fig. S4C). These data match the morphological analysis and suggest that VISTA maintains a more regulatory, homeostatic microglia phenotype in the healthy CNS but does not regulate the microglia LPS response.

To visualize the effect of LPS on gene expression in VISTA KO compared to WT mice, a 4-way plot was generated (Fig. 3c). Genes that were up- or downregulated in both WT and KO after LPS are indicated by gray dots (Fig. 3c). Orange dots represent genes uniquely up- or downregulated in VISTA KO microglia after LPS, and blue dots are genes uniquely up- and downregulated in WT microglia (Fig. 3c). Although no DEGs were found after LPS comparing VISTA KO to WT microglia (Fig. 3a), VISTA KO microglia upregulated 505 genes and downregulated 931 genes which were unaltered in WT microglia (Fig. 3c). The high number of unique DEGs in VISTA KO microglia suggests a distinct transcriptional response to LPS compared to WT. Genes uniquely up- and downregulated in VISTA KO microglia after LPS were lower and higher expressed, respectively, in PBS-treated VISTA KO microglia compared to WT (Fig. 3d). After LPS exposure, these unique DEGs were expressed at equal levels in VISTA KO versus WT microglia. Thus, these genes are uniquely up- and downregulated in VISTA KO due to differences in expression levels at baseline (PBS mice) compared to WT, and not due to differences in induction or depletion of those genes after LPS.

To assess transcriptional co-expression differences between VISTA KO and WT, weighted gene co-expression network analysis (WGCNA) was used. WGCNA resulted in 37 gene modules, of which 7 significantly correlated with LPS treatment, and 2 with genotype (steelblue and paleturquoise) (Additional file 5: Fig. S4D). The Module Eigengene (ME) of the steelblue module was higher expressed in VISTA WT (PBS and LPS) microglia than in VISTA KO microglia (both PBS and LPS) (Fig. 3e) Genes in this module were associated with IL2 and IL6 signaling and other immune processes (Additional file 5: Fig. S4E). The ME of the turquoise module was highest expressed in VISTA KO PBS-treated microglia (Fig. 3e) and genes in this module were annotated with cell cycle processes (Additional file 5: Fig. S4E).

Next, we determined expression of gene sets associated with distinct microglia functions or microglia activation states (Additional file 1: Table S3). VISTA KO microglia exhibited decreased expression of homeostatic microglia markers such as *Cx3cr1, Tmem119,* and *P2ry12*, and sensome genes, a group of genes associated with microglia immune-sensing properties (Fig. 3). Expression of genes associated with proliferating and certain immune-activated (ARM: activated response microglia, DAM: disease-associated microglia, and IFN) microglia was increased [24, 28, 29, 31, 42] (Fig. 3f).

Our data suggest that VISTA is involved in microglia cell cycle functions and induces a more regulatory, homeostatic microglia phenotype in healthy CNS; however, VISTA is not associated with the microglia response to LPS.

### Microglia VISTA KO does not affect EAE induction or progression

Previous results demonstrated that VISTA expression is differentially regulated in MS lesions, but that VISTA KO does not influence microglia response to an acute inflammatory stimulus (LPS). To further dissect the function of VISTA in microglia during neuroinflammation and MS, the effect of VISTA KO on EAE disease development and progression and on microglia transcriptional changes during EAE was assessed.

**Fig. 4:**
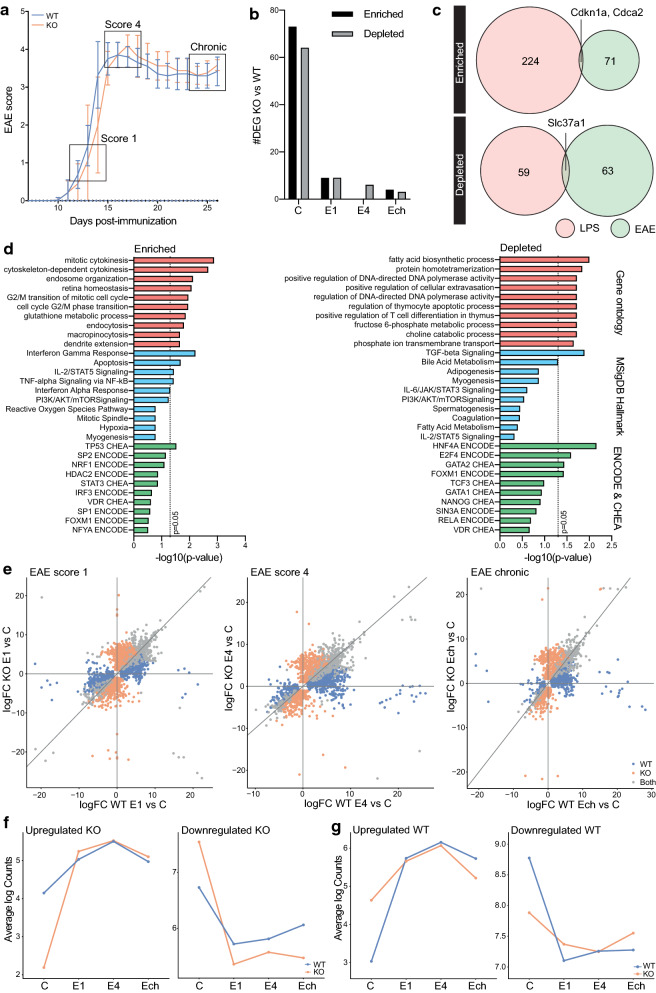
Microglia VISTA KO does not affect EAE progression and induces distinct microglia transcriptional profiles in spinal cord compared to brain. **a** EAE disease progression of VISTA WT and KO mice. Mice were terminated at score 1 (E1: early disease), score 4 (E4: peak disease), and chronic EAE (Ech) and microglia were isolated from spinal cord for mRNA sequencing analysis. **b** Number of differentially expressed genes (DEGs; logFC > 1; adjusted *p*-value < 0.05) comparing VISTA KO vs WT microglia from spinal cord at different EAE stages and in unimmunized mice. **c** Venn diagrams illustrating overlap in enriched (top) and depleted (bottom) genes in VISTA KO compared to WT microglia from PBS-treated mice in LPS experiment (brain; red) and EAE experiment (spinal cord; green). **d** Gene ontology biological processes (red bars), molecular signatures database hallmark (blue bars), and ENCODE/CHEA transcription factors (green bars) associated with genes enriched (left) and depleted (right) in VISTA KO spinal cord microglia compared to WT in unimmunized mice. **e** Four-way plot illustrating logFC of DEGs at different EAE stages compared to unimmunized control in VISTA WT and KO mice. Color of dots indicates whether genes are differentially expressed (adjusted *p*-value < 0.05) in WT (blue), KO (orange), or both (grey). **f–g** Average expression of genes uniquely upregulated (left) or downregulated (right) in VISTA KO **(f)** or WT **G** microglia at all stages of EAE across all samples

Mice were terminated at three different stages of EAE at score 1 (E1: early disease), score 4 (E4: peak disease), and chronic EAE (Ech), and unimmunized mice served as a control (C) (Fig. 4a). Genome-wide transcriptional changes of fluorescence-activated cell sorting (FACS)-isolated spinal cord VISTA KO microglia during EAE were assessed. VISTA depletion using tamoxifen was highly effective at all stages of EAE (Additional file 6: Fig. S5A-B). Furthermore, EAE induced a microglia response which was consistent with previously published microglia EAE profiles (Additional file 6: Fig. S5C-D) [48]. In line with the findings after LPS injection, the induction of microglia EAE genes was highly similar in VISTA KO and WT microglia (Additional file 6: Fig. S5D). Concordantly, EAE disease progression was not affected by VISTA KO (Fig. 4a) and the majority of DEGs comparing VISTA KO and WT were detected in unimmunized mice (Fig. 4b). Only a limited number of these DEGs between WT and VISTA KO spinal cord microglia in unimmunized mice overlapped with DEGs in WT and VISTA KO brain microglia in PBS-treated mice from the previous dataset (Fig. 4c). This limited overlap indicates distinct functional consequences of VISTA KO on spinal cord compared to brain microglia in the healthy CNS. Although VISTA KO led to distinct DEGs in spinal cord compared to brain microglia, the annotated biological processes were similar (Fig. 4d). Genes enriched in spinal cord VISTA KO microglia were associated with cell cycle processes, IFNg, and TNF signaling, whereas depleted genes were annotated with TGFbeta signaling, fatty acid biosynthesis, and other processes (Fig. 4d).

VISTA KO led to an up- and downregulation of distinct sets of genes after LPS injection, which was due to baseline differences as described above (Fig. 3c–d). To assess the effect of EAE on gene expression in VISTA KO microglia compared to WT in a similar manner, a 4-way plot was generated for each EAE stage (Fig. 4e). Grey dots indicate genes differentially expressed in both VISTA KO and WT microglia, orange dots represent genes uniquely differentially expressed in VISTA KO microglia, and blue dots are genes uniquely differentially expressed in WT microglia (Fig. 4e). VISTA KO spinal cord microglia up- and downregulated distinct genes at different stages of EAE compared to WT microglia (Fig. 4e). The majority of genes uniquely up- and downregulated in VISTA KO microglia during EAE did not overlap with genes uniquely up- and downregulated in VISTA KO microglia after LPS injection (Additional file 6: Fig. S5E). Genes uniquely upregulated in VISTA KO microglia during all stages of EAE were depleted in VISTA KO microglia from unimmunized mice, whereas genes uniquely downregulated during EAE were enriched in VISTA KO microglia (Fig. 4f). These results suggest that activation of distinct transcriptional programs during EAE are due to baseline differences of VISTA KO microglia in unimmunized mice, rather than differences in transcriptional profiles of VISTA KO microglia during EAE. Supporting this notion, genes that were uniquely downregulated during EAE in VISTA WT microglia were depleted in unimmunized VISTA KO microglia, whereas expression of genes uniquely upregulated in VISTA WT microglia during EAE was already high in VISTA KO microglia from unimmunized mice (Fig. 4g). Thus, spinal cord VISTA KO microglia already exhibited an EAE-like transcriptional profile in unimmunized mice.

Most sets of genes associated with distinct microglia functions that were higher or lower expressed in VISTA KO brain microglia were not differentially expressed in VISTA KO spinal cord microglia such as homeostatic and proliferating genes (Additional file 6: Fig. S5F). However, spinal cord VISTA KO microglia still had elevated expression of genes associated with interferon microglia (both in C and E1), and sensome genes (both in E1 and E4) (Additional file 6: Fig. S5F). Of note, WGCNA analysis did not yield any modules significantly correlated with genotype (Additional file 6: Fig. S5G).

In summary, VISTA KO does not affect EAE disease progression or microglia transcriptional signatures during EAE. However, as VISTA KO spinal cord microglia possess a distinct transcriptional profile at baseline (unimmunized mice) compared to WT microglia, VISTA KO microglia up- and downregulate distinct sets of genes during EAE. These findings suggest that the impact of VISTA KO on microglia is region-dependent as distinct gene sets are enriched and depleted in VISTA KO microglia from spinal cord and brain. Although these gene sets did not overlap between spinal cord and brain microglia, genes were associated with similar biological processes including cell cycle and immune processes.

### VISTA regulates myelin uptake in microglia, but not phagocytosis of apoptotic cells and *E. coli* particles

VISTA KO affects transcriptional profiles of brain and spinal cord microglia in healthy CNS and thus may perturbate microglia functions that are essential for CNS homeostasis. In macrophages, VISTA regulates the uptake of apoptotic cells (efferocytosis) [13, 49], and in the CNS microglia are important in removing cellular and molecular debris to maintain homeostasis [39]. To assess whether VISTA is involved in microglia efferocytosis or phagocytosis, VISTA was depleted in primary neonatal mouse microglia followed by addition of pHrodo-labelled phagocytic compounds.

Incubation of Cx3cr1^creERT2/WT^ VISTA^loxP/loxP^ primary neonatal mouse microglia with 4-hydroxytamoxifen reduced VISTA expression by up to 90% at the mRNA and protein level (= VISTA KO) compared to ethanol-treated cells (= VISTA WT) (Additional file 7: Fig. S6A-B). Treatment of microglia with 4-hydroxytamoxifen did not induce *Tnf* expression (Additional file 7: Fig. S6C).

**Fig. 5:**
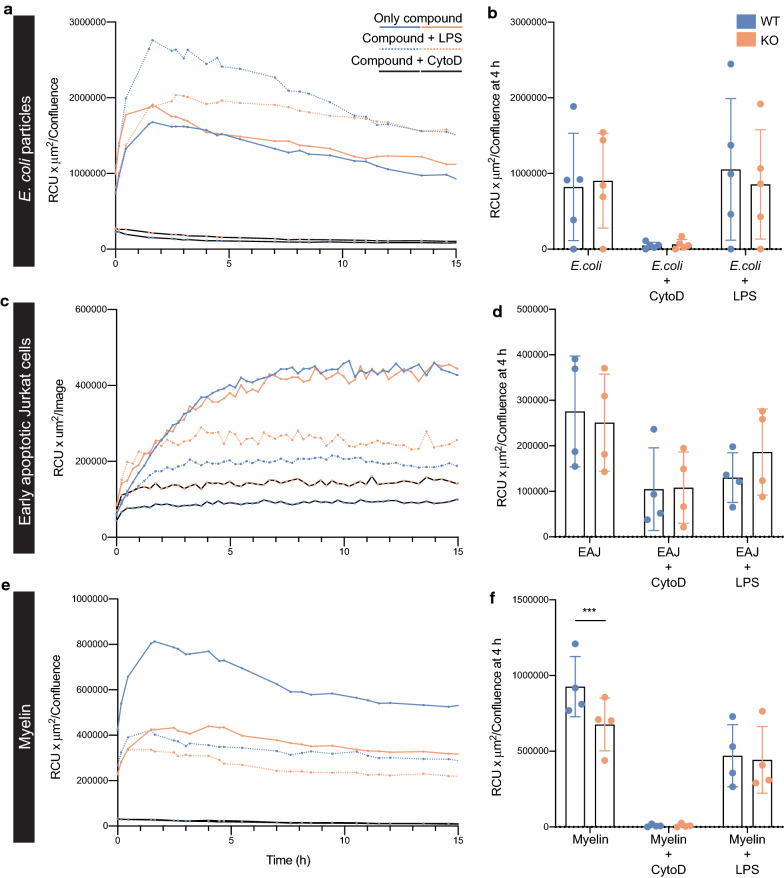
VISTA KO in microglia impairs myelin phagocytosis but not uptake of other particles**.** VISTA was depleted in Cx3cr1^creERT2/WT^ VISTA^loxP/loxP^ primary neonatal mouse microglia using 4-hydroxytamoxifen followed by incubation with pHrodo-labelled compounds. **a,c,e** Representative graphs of fluorescence intensity of pHrodo-labelled *E. coli* particles **a**, early apoptotic Jurkat cells **c**, and mouse myelin **e** depicted as red calibrated unit (RCU) plotted against time after addition of compounds and normalized by confluence (n = 4–5). **b,d,f** Fluorescence intensity normalized to confluence after 4 h incubation with pHrodo-labelled *E. coli* particles **b**, early apoptotic Jurkat cells **d**, and mouse myelin **f.** Error bars indicate mean ± s.d. (n = 4–5). Statistical analysis performed was a 2-way ANOVA with Sidak’s correction for multiple comparisons comparing KO to WT. ***p < 0.001, CytoD = Cytochalasin D, EAJ = Early apoptotic Jurkat cells

Depletion of VISTA did not alter the uptake of pHrodo-labelled *E. coli* particles, in line with published findings in macrophages [49]. Jurkat cell apoptosis was induced using staurosporine and after 4 h, more than 90% of Jurkat cells were early apoptotic (Additional file 7: Fig. S6D). Efferocytosis of early apoptotic Jurkat cells was unaltered in VISTA KO microglia (Fig. 5c–d). Next, VISTA KO primary neonatal mouse microglia were incubated with the CNS component myelin. A ~ 25% reduction in myelin uptake was observed in VISTA KO microglia compared to WT (Fig. 5e–f). LPS preconditioning reduced the uptake of myelin by ~ 50%, but there was no difference between VISTA KO and VISTA WT microglia (Fig. 5e–f).

These data demonstrate that VISTA depletion in microglia in vitro does not affect uptake of *E. coli* particles or efferocytosis of early apoptotic Jurkat cells, but reduces uptake of myelin.

## Discussion

Here, we demonstrate differential VISTA expression in MS lesion stages and present an in-depth analysis of potential VISTA function in microglia during health and neuroinflammation. VISTA function has extensively been studied in T cells and myeloid cells of the periphery, but not in microglia. Collectively, our current data demonstrate that VISTA function in microglia is distinct from that in macrophages, since VISTA KO affects expression of genes associated microglia proliferation, immune-activation, and VISTA KO in vitro affects microglia phagocytic ability.

VISTA expression was increased in MS lesions that presented with inflammation (high HLA-DR expression), but was decreased in MS lesions that lacked inflammation. In contrast, microglia VISTA is decreased in CNS disease mouse models that involve microglia activation including EAE, cuprizone, neurodegenerative diseases, and other neuroinflammatory models [6, 7]. MS lesions are highly heterogeneous regarding their pathophysiology and criteria used to stage these lesions include degree of demyelination and inflammation [32, 43, 44]. MS mouse models such as EAE do not recapitulate the heterogeneity of MS lesions and are very acute in contrast to MS as a chronic disease. Thus, it is possible that these deviating observations in mouse and humans are due to a more complex environment in human MS. Furthermore, differences in VISTA could be explained by species differences. It is also possible that other cell types found in active MS lesions such as peripheral immune cell subsets contribute to VISTA expression, or that other CNS resident cell types upregulated VISTA during MS.

VISTA could be involved in MS pathology due to its role in inhibition of T-cell activation, the maintenance of a homeostatic microglia phenotype, and the regulation of microglia myelin uptake. In the context of MS, an enhanced expression of VISTA in immunologically active lesions can potentially be beneficial since infiltrating T cells could become inactivated. In view of VISTA function in maintaining a more homeostatic microglia phenotype in naïve mice, and upregulation of VISTA in preactive and active lesions may also serve as an effort to avoid aberrant microglia activation. Decreased VISTA expression in inactive lesions might reflect a reduction in microglia numbers, as VISTA more strongly correlated with expression of microglia markers (IBA1 and TMEM119) compared to inflammatory markers (HLA-DR and CD68). Interestingly, VISTA KO in microglia in vitro reduced the uptake of myelin, which may have consequences for MS lesion pathology. In MS, uptake of intact myelin may contribute to disease by damaging oligodendrocytes, axons, and presenting brain antigens to infiltrating T cells [23]. However, clearance of myelin debris is beneficial in MS since this promotes tissue repair and induces a more regulatory myeloid phenotype by releasing anti-inflammatory mediators such as IL10, CCL18, and prostaglandin-E 2 (PGE2) [8, 23, 53]. Therefore, microglia VISTA in MS lesion stages potentially regulates myelin uptake, thereby contributing to oligodendrocyte damage, antigen-presentation, but also tissue repair, and induction of a more regulatory, homeostatic microglia phenotype. Since VISTA has a potential role in MS pathogenesis, a therapeutic intervention targeting VISTA is conceivable, and we extensively discussed the therapeutic potential of VISTA in MS previously [7].

Of note, VISTA deficiency in microglia in vitro led to a decreased uptake of myelin, but not of early apoptotic Jurkat cells or *E. coli* particles. In macrophages, VISTA is involved in the uptake of apoptotic thymocytes [49] and neutrophils [13], but not beads or *E. coli* particles [49]. It is unlikely that uptake of early apoptotic Jurkat cells requires distinct pathways from uptake of apoptotic thymocytes and neutrophils. Thus, VISTA likely regulates different phagocytosis pathways in microglia compared to macrophages.

To assess whether increased or decreased VISTA expression in MS and various mouse models of neuroinflammation has consequences for disease development, we characterized EAE progression and transcriptional profiles of VISTA KO microglia after LPS and EAE. Generic VISTA KO increases the susceptibility to developing autoimmunity and inflammatory diseases including murine lupus nephritis [11], imiquimod-induced psoriasis [35], systemic lupus erythematosus [25], experimental asthma [36], and EAE [46]. In contrast, our current study demonstrates that microglia VISTA KO did not affect EAE induction or progression. In addition, there was no difference in transcriptional profiles of VISTA KO compared to WT mice after LPS exposure and during EAE. VISTA is widely expressed by immune cell types in the periphery including T cells and myeloid cells [17] and thus VISTA expressed on peripheral immune cells and not microglia is likely involved in the development and progression of EAE. Generic VISTA KO mice present with a more activated myeloid cell compartment as evident from increased cytokine and chemokine production [9, 35], which likely exacerbates autoimmune and inflammatory diseases in mice. The CNS environment differs from the periphery as immune responses and interactions especially with T cells are more limited. Due to limited regenerative potential of the CNS, microglia immune responses must be more tightly regulated than those of peripheral macrophages as to avoid excessive CNS tissue damage. It is possible that in contrast to macrophages, VISTA KO does not affect microglia under inflammatory conditions because of a more stringent regulation of microglia immune responses.

Although VISTA KO did not affect microglia responses to LPS and during EAE, we report substantial morphological and transcriptional alterations in VISTA KO microglia compared to WT during homeostasis. VISTA KO microglia exhibited a more amoeboid morphology which is associated with an immune-activated microglia profile during neuroinflammation [16]. In accordance with these morphological observations, VISTA KO induced a more immune-activated microglia profile, demonstrated by upregulation of genes associated with TNF and IFN signaling, and by an upregulation of gene sets associated with microglia in neurodegenerative disease and interferon response. Thus, microglia VISTA might induce a more regulatory, homeostatic microglia phenotype. Similarly, VISTA maintains quiescence in peripheral myeloid cells [18]. Activation of VISTA using agonistic antibodies induces a more regulatory profile in macrophages [18]. Homeostatic microglia functions are essential for the developing CNS and for maintaining a healthy adult CNS as microglia are involved in clearing cellular and molecular debris, synaptic pruning, and secrete neurotrophic factors [39]. It is conceivable that a perturbation in microglia homeostatic functions due to VISTA KO may compromise CNS homeostasis. No behavioral tests were performed on microglia VISTA KO mice and thus the effect of this VISTA KO-induced immune-activated microglia phenotype on the healthy CNS remains to be elucidated. Furthermore, VISTA KO microglia were enriched for genes involved in cell cycle, a potential function for VISTA that has not been reported for peripheral myeloid cells. Although genes involved in apoptotic pathways were not differentially expressed in VISTA KO microglia, VISTA KO may reduce survivability of microglia which may partially be rescued by upregulation of cell cycle genes. A low percentage of VISTA^pos^ microglia (< 2%) escaped tamoxifen-induced *VISTA* excision and if VISTA KO reduced the life span of microglia, the frequency of VISTA^pos^ microglia should increase at late time points after tamoxifen administration. However, we did not observe an increase in VISTA^pos^ microglia from 1.5 to 6 months after tamoxifen administration. Hence, it is more likely that VISTA directly regulates the expression of cell cycle genes in microglia and might be involved in microglia proliferation.

Collectively, our data provide novel insights that VISTA regulates microglia myelin uptake, proliferation, and imparts a more regulatory, homeostatic microglia phenotype. Although VISTA KO does not affect the inflammatory response of microglia after LPS exposure and during EAE, a dysregulation of microglia homeostasis may perturb the healthy CNS. Impaired uptake of myelin may also have consequences for MS lesion progression as myelin debris clearance is an important part of tissue regeneration. In conclusion, we present multiple potential functions of VISTA in microglia biology which could extend to perturbed CNS homeostasis and might contribute to development and progression of neuroinflammatory diseases. VISTA is amenable to clinical manipulation with agonist and antagonist biologicals which are under (pre)clinical development.

## Supplementary Information

**Additional file 1**. Extended data containing Tables S1-S5. Table S1. Information on MS patients. Table S2. Morphometric features of individual cells. Table S3. Genes in published microglia gene sets. Table S4. Normcounts and differential gene expression in LPS experiment. Table S5. Normcounts and differential gene expression in EAEexperiment

**Additional file 2**. **Fig. S1. MS lesion staging based on PLP and HLA-DR expression**. Representative images of PLP and HLA-DRexpression which were used for lesion staging based on degree of demyelination (PLP) and inflammation (HLA-DR) [32, 43, 44].

**Additional file 3**. **Fig. S2. VISTA expression in MS lesion stages correlates with expression of microglia markers IBA1 and TMEM119**. (**A**–**C**) Representative images of in situ expression of IBA1 (**A**), TMEM119 (**B**), and CD68 (**C**) in MS lesion. (**D**–**F**) Quantification of positive areas of IBA1 (D), TMEM119 (E), and CD68 (**F**) in MS lesions. At least 3 images were randomly selected from each lesion and the area positive for VISTA was measured. Boxes indicate median and lower and upper quartiles, whiskers show min/max, and outliers are indicated by points (n=5 for each lesion). Statistical analysis performed was a Wilcoxon signed-rank test comparing lesions to normal-appearing areas with Benjamini–Hochberg correction for multiple comparisons. (**G**) Correlation of CD68, HLA-DR, TMEM119, IBA1, and VISTA expression in all MS lesion stages using linear regression. ns = not significant, *p < 0.05, **p < 0.01, ***p < 0.001, ****p < 0.0001

**Additional file 4**. **Fig. S3. Conditional VISTA KO in microglia is stable and effective**. (**A**–**B**) VISTA surface expression (**A**) and mRNA expression (**B**) in microglia (DAPI^neg^ CD11b^pos^ CD45^int^ Ly6C^neg^) isolated 1.5 months, 3 months, and 6 months after oil or tamoxifen treatment of Cx3cr1^creERT2/WT^ VISTA^loxP/loxP^ mice. Error bars indicate mean ± s.d. (n=3–4). Statistical analysisperformed was a one-way ANOVA with Bonferroni correction for multiple comparisons. (**C**) VISTA surface expression in WT and VISTA KO brain microglia (DAPI^neg^ CD11b^pos^ CD45^int^ Ly6C^neg^) after oil or tamoxifen treatment respectively and after PBS or LPS injection. Error bars indicate mean ± s.d. (n=5). Statistical analysis performed was a one-way ANOVA with Bonferroni correction for multiple comparisons.

**Additional file 5**. **Fig. S4. VISTA KO does not affect the induction of microglia LPS response genes, and two WGCNA modules correlate with genotype**. (**A**) PCA of VISTA WT and KO microglia after PBS and LPS injection. (**B**) Average expression of our published gene set upregulated in microglia after LPS [22] across all samples. (**C**) LogFC of Tnf (left) and Ccl2 (right) expression in primary neonatal mouse microglia after stimulation with various inflammatory mediators comparing tamoxifen-treated (VISTA KO) and ethanol-treated (VISTA WT) microglia. Error bars indicate mean ± s.d. (n=2–5). Statistical analysis performed was a one sample t test. (**D**) Correlation of Module Eigengenes with genotype (VISTA KO-WT) and condition (LPS-PBS). Color indicates Pearson R and numbers indicate *p*-value. (**E**) Gene ontology biological processes (red bars), molecular signatures database hallmark (blue bars), and ENCODE/CHEA transcription factors (green bars) associated with genes in WGCNA modules that significantly correlate with genotype: steelblue (left) and paleturquoise (right).

**Additional file 6**. **Fig. S5. VISTA KO efficiency in spinal cord microglia and effects of VISTA KO on microglia EAE signature**. (**A**–**B**) Percentage of VISTA microglia (DAPI^neg^ CD11b^pos^ CD45^int^ Ly6C^neg^) (**A**) and geometric mean fluorescence intensity of VISTA in microglia (**B**) in spinal cord VISTA WT and KO microglia during EAE. Error bars indicate mean ± s.d. (n=5). Statistical analysis performed was a two-way ANOVA with Sidak’s correction for multiple comparisons. ****p < 0.0001 (**C**) PCA of VISTA WT and KO microglia at different stages of EAE. (**D**) Average expression of published gene sets upregulated in microglia after EAE [48] across all samples. (**E**) Venn diagram illustrating overlap in uniquely upregulated (top) and downregulated (bottom) genes in VISTA KO microglia after LPS (brain; red) and during all stages of EAE (spinal cord; green). (**F**) Correlation of module Eigengenes with genotype (VISTA KO-WT) and condition (EAEstages). Color indicates Pearson R and numbers indicated *p*-value. (**G**) Average expression of published gene sets associated with distinct microglia functions across all samples [24, 28, 29, 31, 42]. Information of gene sets can be found in Table S3.

**Additional file 7**. **Fig. S6. VISTA KO efficiency in primary neonatal mouse microglia and apoptosis induction in Jurkat cells**. (**A**, **C**) VISTA (**A**) and Tnf (**C**) mRNA expression in Cx3cr1^creERT2/WT^ VISTA^loxP/loxP^ primary neonatal mouse microglia after 24 h incubation with different concentrations of 4-hydroxytamoxifen and 24 h resting period measured by RT-qPCR and normalized to Hprt1. Error bars indicate mean ± s.d. (n=1–4). Statistical analysis performed was a one-way ANOVA with Dunnett’s correction for multiple comparisons comparing 4-hydroxytamoxifen-treated groups to EtOH control. (**B**) VISTA surface expression on Cx3cr1^creERT2/WT^ VISTA^loxP/loxP^ primary neonatal mouse microglia after 24 h incubation with different concentrations of 4-hydroxytamoxifen and 24 h resting period (n=2). (**D**) Percentage of alive (AnnexinV^neg^/PI^neg^), early apoptotic (AnnexinV^pos^/PI^neg^), and late apoptotic/necrotic (AnnexinV^pos^/PI^pos^) Jurkat cells after 1–4 h incubation with 1 μM staurosporine and representative FACS plot of Jurkat cells after 4 h staurosporine incubation. *p < 0.05, **p < 0.01, ***p < 0.001, ****p < 0.0001, EtOH = Ethanol, Tam = 4-hydroxytamoxifen, PI = Propidium iodide
